# Impact of hypertension on long-term humoral and cellular response to SARS-CoV-2 infection

**DOI:** 10.3389/fimmu.2022.915001

**Published:** 2022-09-02

**Authors:** Chang Chu, Anne Schönbrunn, Kristin Klemm, Volker von Baehr, Bernhard K. Krämer, Saban Elitok, Berthold Hocher

**Affiliations:** ^1^ Fifth Department of Medicine (Nephrology/Endocrinology/Rheumatology/Pneumology), University Medical Centre Mannheim, University of Heidelberg, Heidelberg, Germany; ^2^ Department of Nephrology, Charité - Universitätsmedizin Berlin, Berlin, Germany; ^3^ Institute of Medical Diagnostics (IMD) Berlin-Potsdam, Berlin, Germany; ^4^ Department of Nephrology and Endocrinology, Ernst von Bergmann Hospital Potsdam, Potsdam, Germany; ^5^ European Center for Angioscience (ECAS), Faculty of Medicine of the University of Heidelberg, Mannheim, Germany; ^6^ Center for Preventive Medicine and Digital Health Baden-Württemberg, Center for Preventive Medicine and Digital Health Baden-Württemberg (CPDBW), Medical Faculty Mannheim, Heidelberg University, Mannheim, Germany; ^7^ Mannheim Institute for Innate Immunoscience, Medical Faculty Mannheim of the University of Heidelberg, Mannheim, Germany; ^8^ Key Laboratory of Study and Discovery of Small Targeted Molecules of Hunan Province, School of Medicine, Hunan Normal University, Changsha, China; ^9^ Reproductive and Genetic Hospital of China International Trust Investment Corporation (CITIC)-Xiangya, Changsha, China

**Keywords:** hypertension, humoral and cellular response, SARS-CoV-2 infection, COVID - 19, immune response

## Abstract

It was shown that hypertension delays SARS CoV-2 viral clearance and exacerbates airway hyperinflammation in the respiratory tract. However, it is unknown whether hypertension determines the long-term cellular and humoral response to SARS Cov2. Health care workers (HCWs) after an outbreak of SARS Cov-2 infections were analyzed. Infected HCWs were not vaccinated before blood collection. 5-14 months (median 7 months) after detection of SARS CoV-2 infection, blood was taken to analyze humoral response (S1 IgG and SARS CoV-2 neutralizing antibodies) and cellular (T cell responses to SARS-CoV-2 with Lymphocyte Transformation Test). To identify clinical factors that determine the immune response, a multivariate regression analysis was done considering age, BMI, sex, diabetes, hypertension, smoking, COPD, asthma and time between PCR positivity and blood collection as confounding factors. Infected hypertensive HCWs more often needed to be hospitalized than non-hypertensive HCWs, but were less likely to develop anosmia and myalgia. The long-term humoral and cellular immune response was significantly strengthened in hypertensive versus normotensive infected HCWs. Multivariate regression analysis revealed that hypertension was independently associated with the humoral response to SARS CoV-2 infection. Multivariate regression analysis using same confounding factors for the humoral response showed a clear trend for an association with the cellular response to SARS CoV-2 infection as well. In conclusion, SARS CoV-2 infection strengthened immune response to SARS CoV-2 infection in hypertensive HCWs independent of other risk factors.

## Introduction

Infections with the SARS Cov-2 virus led to cellular and humoral responses of the human immune system (1). Systematic comparisons of the humoral and cellular response of the immune system after SARS CoV-2 infection and vaccination in well-defined populations are necessary to better understand clinical factors that regulate the humoral and cellular response of the immune system after infection and vaccination, respectively. We did this by following staff at a hospital where a severe COVID-19 outbreak occurred. The COVID-19 outbreak at Potsdam’s Ernst von Bergmann Hospital (EvB) made headlines across Germany. In March and April 2020, 47 people infected with SARS CoV-2 died at the municipal hospital. According to the hospital, 44 of the cases were patients who had not come to the hospital because of a SARS Cov-2 infection, but with a different diagnosis. This has probably been the most severe corona outbreak in a German hospital. After the outbreak, the hospital’s hygiene measures were tightened. All staff members were given a nasal-oral swab twice a week thereafter. The swab material was analyzed by PCR for possible infections with the SARS CoV-2 virus.

The aim of this study was to understand which clinical factors in a middle-aged European health care worker population (employees of the Ernst von Bergmann Hospital, Potsdam, Germany) influence the long-term humoral and cellular response to SARS CoV-2 infection or vaccination. We were particularly interested in the impact of hypertension on the long-term humoral and cellular immune response to SARS CoV-2 virus infection, since it was recently shown that hypertension delays viral clearance and exacerbates airway hyperinflammation in patients with COVID-19 (2).

## Methods

### Study population

After the COVID-19 outbreak in the Ernst von Bergmann Hospital, Potsdam, Germany, in March and April 2020, all health care workers (HCWs) were given a nasal-oral swab analyzed by PCR for possible SARS CoV-2 infections twice a week.

Sixteen months later, we offered staff who had been infected and not yet vaccinated to examine the humoral and cellular response after infection. The infection had to have occurred at least 4 months prior. We also recruited a second group of employees who had not been infected during the observation period and were fully vaccinated (two doses of a COVID-19 vaccine approved in Germany) against SARS CoV-2 between May and July 2021. Blood was drawn from these subjects 21 to 195 days after vaccination. All study participants were examined by study physicians. The following parameters were recorded: age, sex, BMI, type 1 or 2 diabetes (yes/no), hypertension (hypertensive blood pressure levels according to the European Society of Hypertension guidelines (3) or drug treatment of known hypertension) (yes/no), smoking (yes/no), COPD (yes/no) and asthma (yes/no). We recorded the time from PCR detection of SARS CoV-2 infection to blood collection and the time from complete vaccination to blood collection. We also recorded, whether the subjects became symptomatic after infection or whether vaccination side effects were observed. The study was approved by the ethical committee of the association of physicians in the province of Brandenburg, Germany. All study participants gave their written, informed consent to the study.

### Measurement of T cell responses to SARS-CoV-2 with lymphocyte transformation test (lymphocyte proliferation test)

Heparinized venous blood was processed by density gradient centrifugation to obtain peripheral blood mononuclear cells (PBMCs). After washing the cells twice with PBS (SIGMA-Aldrich), cell pellet was resuspended to obtain a cell count of 1 x 10^6^/ml in cell culture medium (RPMI 1640; Biowest) supplemented with 2 mM L-glutamine, 100 μg/ml gentamicin (all from Biowest) and 5% autologous serum. Specific T cell reactions were assessed by a lymphocyte proliferation assay (LTT). Therefore, 2 x 10^5^ PBMCs were either incubated with peptide pool 1 or 2 of SARS-CoV-2 spike glycoprotein (PM-WCPV-S from JPT) using a concentration of 1µg/ml per peptide, together with 1µg/ml anti-CD28 Abs (clone CD28.2 from BD Biosciences). Both pools contained 15-mer peptides that overlapped 11 amino acids, respectively and in total spanned the entire SARS-CoV-2 spike glycoprotein. The N-terminal part, containing the RBD-region, was covered by pool 1 (N-Term) and the C-terminal part of the protein was covered by pool 2 (C-Term). Detailed information about the peptide pools have been given before (4). Two positive controls were performed by stimulating cells with a mixture of recall-antigens, containing tetanus, influenza and candida albicans (antigen control) as well as with pokeweed mitogen (mitogen control). For base level control cells were left unstimulated. All stimulations were performed in triplicates in a 96-well plate for 5 days at 37°C and 5% CO_2_ atmosphere. Cells were labeled with 3H-thymidine (1 μCi/ml, HARTMANN ANALYTIC) 12 hours prior to cell harvest. A cell harvester (PerkinElmer) was used to harvest cells on glass fiber filters. The incorporated 3H-thymidine activity was measured as “counts per minute” (cpm) using a solid phase beta counter (PerkinElmer). For analysis mean values of the triplicates were calculated. The results for each stimulation were finally given as a stimulation index (SI; ratio of cpm of cell culture with and without stimulation). A SI ratio of >2 was considered as a positive SARS-CoV-2 lymphocyte transformation test, because in a control cohort of 88 patients without any clinical or laboratory evidence for SARS-CoV-2 infection (all criteria needed to be fulfilled in the control cohort: no contacts to patients with proven SARS Cov-2 infection, absence of any symptoms of a SARS Cov-2 infection, no detection of virus RNA in a nasal swob by RT-PCR, no detection of SARS-CoV-2 antibodies), all SI values were below 2 (5).

### Measurement of humoral immune response to SARS-CoV-2

Serum samples were measured for the presence of anti-SARS-CoV-2 IgG using commercial kits. For quantitative detection of IgG against SARS-CoV-2 spike glycoprotein 1 (S1) enzyme-linked immunosorbent assay (ELISA; EUROIMMUN) was performed on an automated ANALYZER system (QuantiVac, EUROIMMUN) according to manufacturer´s instructions. The assay relies on 6 calibrators in order to quantify the IgG (S1)-concentration given as BAU/ml (Binding Antibody Units) and highly correlates with the “First WHO International Standard” (NIBSC code: 20/136). Values between 25.6 and 35.2 BAU/ml are considered to be borderline, values above 35.2 BAU/ml were interpreted as positive. The assay is based on the previously established semi-quantitative assay, which has been already described (6).

### Surrogate virus neutralization test (sVNT)

SARS-CoV-2 sVNT Kit (cPAss from Genscript) was used to evaluate the neutralizing capacity of anti-SARS-CoV-2 antibodies present in the serum. This is a blocking enzyme-linked immunosorbent assay (ELISA), which mimics the virus-host interaction. Binding of a horseradish peroxidase conjugated RBD-fragment of the SARS-CoV-2 (HRP-RBD) to the human host ACE2 receptor can be blocked by neutralizing antibodies against the SARS-CoV-2 spike protein, containing the RBD in the serum or plasma. The strength of HRP signal indicates the degree of blockage and therefore indirect the neutralizing capacity. The sVNT assay from Genscript has been validated and described previously (7–10).

### Statistical analysis

Descriptive variables are shown as medians (interquartile ranges) or numbers (percentage). Comparisons were assessed by Mann-Whitney U test, or χ^2^ test, as appropriate. Spearman correlation analysis was performed to assess correlation for humoral and cellular response parameters. Multivariate linear regression analysis was performed with wild bootstrapping to determine the humoral and cellular response coefficients of the 9 candidate factors in infected or vaccinated HCWs, i.e., age (years), sex (M/F), body mass index (BMI), diabetes (yes/no), hypertension (yes/no), COPD (yes/no), Asthma (yes/no), smoking (yes/no), time from infection/vaccination to blood collection. In vaccinated HCWs, the type of vaccinations was extra added in the model. All statistical analyses were performed using SPSS 25.0 software (SPSS, Chicago, IL, USA). The level of significance was set at p<0.05.

## Results

### Study population

268 HCWs were detected by the twice weekly PCR testing as positive after the COVID-19 outbreak. 180 HCWs got at least one dose of a COVID-19 vaccine approved in Germany. 71 HCWs were fully vaccinated at time of blood taking. We asked either the infected but not vaccinated HCWs (n=88) and the fully vaccinated but not infected HCWs (n=71) to participate in our study. Among 71 fully vaccinated HCW, 37 had AstraZeneca, 31 had BioNTech, 2 had Moderna as the first shot, 47 had BioNTech, 19 had AstraZeneca, 4 had Moderna as the second shot, one of 71 HCWs had Johnson. No significant statistical difference in anti-hypertensive treatment between hypertensive HCWs in the infected group and hypertensive HCWs in the vaccinated group.

Clinical data, humoral and cellular immune response of SARS-CoV-2 infected HCWs are shown in **Table 1**. Males comprised 34.1% of the cohort (30 males, 58 females). Underlying health conditions were diabetes in 8 cases (9.1%), hypertension in 25 cases (28.4%), COPD in 2 cases (2.3%), asthma in 9 cases (11.5%). Blood was taken at a median of 6.8 (4.8, 14.4) months after SARS-CoV-2 infection proven by validated RT-PCR. Clinical data, humoral and cellular immune response parameters of vaccinated HCWs, and comparisons between vaccinated HCWs with and without hypertension are shown in **Table 2**. Blood was taken at a median of 2.0 (1.0, 2.0) months after vaccination. **Table 3** provides data on symptoms and duration between infection and blood taking of infected HCWs, shows that infected hypertensive HCWs more often needed to be hospitalized than non-hypertensive HCWs, but were less likely to develop anosmia and myalgia. **Table 4** presents bivariate associations between humoral and cellular response parameters. Measurements of SARS-CoV-2 IgG-Ab (S1) and the SARS CoV-2 surrogate neutralization test showed a strong association (rho=0.887, p<0.0001), as well as LTT Spike-N-Term and Spike-C-Term (rho=0.828, p<0.0001).

**Table 1:** Characteristics of SARS-CoV-2 infected health care workers (HCWs).

Parameters	Infected HCWs (n=88)	Infected HCWs with hypertension (n=25)	Infected HCWs without hypertension (n=63)	p value
Age (years)	46.0 (34.0, 59.0)	60.0 (51.0, 66.0)	41.0 (31.0, 51.0)	p<0.0001
Sex (M/F)	30/58	14/11	16/47	p=0.007
BMI	24.8 (22.8, 28.4)	27.5 (24.4, 32.8)	24.1 (22.7, 26.7)	p=0.006
Diabetes (yes/no)	8/80	7/18	1/62	p=0.0001
Hypertension (yes/no)	25/63	25/0	0/63	-
COPD (yes/no)	2/86	2/23	0/63	p=0.024
Asthma (yes/no)	9/78	3/22	6/56	p=0.749
Smoking (yes/no)	6/82	3/22	3/60	p=0.227
SARS-CoV-2 IgG-Ab (S1) (BAU/ml)	116.0 (47.3, 265.0)	224.0 (79.5, 768,0)	85.9 (39.7, 207.0)	p=0.004
SARS surrogate neutralization test (%)	63.5 (41.0, 88.5)	83.0 (49.8, 94.0)	57.5 (37.3, 83.5)	p=0.009
T cell responses to SARS-CoV-2 Spike-N-Term (SI)	5.4 (2.8, 9.2)	9.1 (4.5, 20.6)	4.1 (2.4, 6.6)	p=0.001
T cell responses to SARS-CoV-2 Spike-C-Term (SI)	4.1 (2.7, 7.8)	7.5 (4.0, 12.0)	3.5 (2.1, 5.1)	p=0.001
Time from infection to blood collection (days)	204 (144, 432)	188 (131, 429)	204 (162, 436)	p=0.266

SARS-CoV-2 infection was confirmed by PCR test. Continuous variables are given as medians (interquartile range) or numbers. Body mass index (BMI) was calculated as weight in kilograms divided by height in meters squared. COPD, chronic obstructive pulmonary disease. Comparison between hypertension and non-hypertension group was performed by Mann-Whitney U test.

**Table 2:** Characteristics of vaccinated health care workers (HCWs).

Parameters	Vaccinated HCWs(n=71)	Vaccinated HCWs with hypertension (n=15)	Vaccinated HCWs without hypertension (n=56)	p value
Age (years)	44.0 (32.0, 55.0)	57.0 (54.0, 65.0)	37.0 (31.3, 50.5)	p<0.0001
Sex (M/F)	21/50	2/13	21/39	p=0.123
BMI	24.9 (22.3, 28.0)	28.4 (25.0, 31.2)	23.7 (21.6, 26.9)	p=0.001
Diabetes (yes/no)	4/67	4/11	0/60	p<0.0001
Hypertension (yes/no)	15/56	15/0	0/60	-
COPD (yes/no)	0/71	0/15	0/60	1.000
Asthma (yes/no)	8/63	3/12	6/54	0.232
Smoking (yes/no)	15/56	4/11	13/47	0.557
SARS-CoV-2 IgG-Ab (S1) (BAU/ml)	768.0 (218.3, 768.0)	233.0 (120.0, 768.0)	768.0 (290.0, 768.0)	0.006
SARS surrogate neutralization test (%)	93.5 (71.0, 97.0)	79.0 (60.0, 96.0)	95.0 (78.0, 97.0)	0.048
T cell responses to SARS-CoV-2 Spike-N-Term (SI)	5.6 (3.4, 8.9)	4.2 (2.1, 5.8)	6.0 (3.9, 9.8)	0.088
T cell responses to SARS-CoV-2 Spike-C-Term (SI)	4.4 (2.6, 6.7)	4.4 (2.2, 7.9)	4.5 (2.8, 6.7)	0.464
Time from vaccination to blood collection (days)	71 (54, 77)	71 (62, 99)	70 (53, 77)	0.747

SARS-CoV-2 infection was confirmed by PCR test. Continuous variables are given as medians (interquartile range) or numbers. Body mass index (BMI) was calculated as weight in kilograms divided by height in meters squared. COPD, chronic obstructive pulmonary disease. Comparison between hypertension and non-hypertension group was performed by Mann-Whitney U test.

**Table 3:** Comparison of baseline symptoms and blood taking duration between SARS-CoV-2 infected patients with and without hypertension.

Parameters	hypertension patients (n=25)	non-hypertension patients (n=63)	p value
Symptom (yes/no)	25/0	57/5	0.316
Anosmia (yes/no)	8/17	36/26	0.034
Headache (yes/no)	8/17	22/40	0.757
Fever (yes/no)	12/13	25/37	0.512
Dyspnea (yes/no)	7/18	8/54	0.092
Myalgia (yes/no)	1/24	15/47	0.032
Hospital-admitted (yes/no)	9/16	3/59	0.0005
Time from infection to blood collection (days)	236.7 ± 146.3	272.1 ± 146.9	0.266

Continuous variables are given as mean ± SD. Data on clinical symptoms of one non-hypertension patients were missing. Comparisons were assessed by χ^2^ test.

**Table 4:** Bivariate associations between humoral and cellular response parameters.

Correlation analysis					
		SARS-CoV-2 IgG-Ab (S1) (BAU/ml)	SARS surrogate neutralization test (%)	Spike-N-TermLTT (SI)	Spike-C-TermLTT (SI)
SARS-CoV-2 IgG-Ab (S1) (BAU/ml)	Correlation Coefficient	-	0.887	0.455	0.370
	Sig. (2-tailed)		<0.0001	<0.0001	<0.0001
SARS surrogate neutralization test (%)	Correlation Coefficient	0.887	-	0.451	0.363
	Sig. (2-tailed)	<0.0001		<0.0001	<0.0001
Spike-N-Term LTT (SI)	Correlation Coefficient	0.455	0.451	-	0.828
	Sig. (2-tailed)	<0.0001	<0.0001		<0.0001
Spike-C-Term LTT (SI)	Correlation Coefficient	0.370	0.363	0.828	-
	Sig. (2-tailed)	<0.0001	<0.0001	<0.0001	

Spearman rank correlation analysis was used to determine the correlations between humoral and cellular response parameters.

### Humoral and cellular immune response to SARS-CoV-2

**Figure 1:**
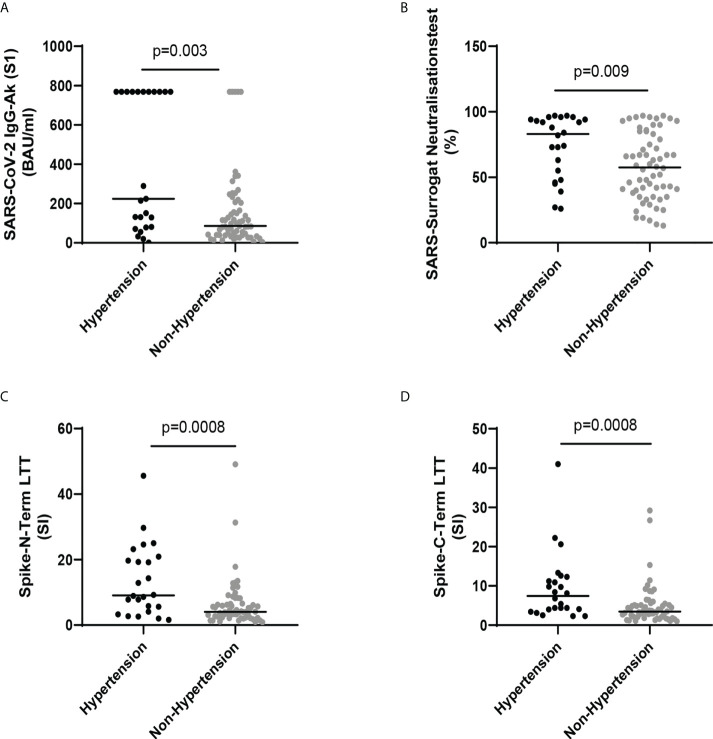
**(A)** SARS-CoV-2 IgG-Ab (S1) (BAU/ml) in SARS-CoV-2 infected health care workers (HCWs) with and without hypertension. **(B)** SARS surrogate neutralization test (%) in SARS-CoV-2 infected HCWs with and without hypertension. **(C)** Spike-N-Term LTT (SI) in SARS-CoV-2 infected HCWs with and without hypertension. **(D)** Spike-C-Term LTT (SI) in SARS-CoV-2 infected HCWs with and without hypertension. Lines show median. Comparison was made by Mann-Whitney U test.

The humoral response to SARS Cov-2 infection in the HCWs was analyzed by measuring SARS-CoV-2 IgG-Ab (S1) and the SARS CoV-2 surrogate neutralization test. Both tests consistently showed markedly enhanced activation of the humoral immune system in infected hypertensive HCWs compared with infected HCWs with normal blood pressure (**Table 1** and **Figure 1**). The cellular response to SARS Cov-2 infection in the HCWs was analyzed by the Lymphocyte Transformation Test (LTT) adding either peptides from the N- or C-terminus of the spike protein to the purified lymphocytes of the patient. This analysis likewise showed a significantly enhanced activation of the specific cellular immune system in hypertensive infected HCWs (**Table 1** and **Figure 1**).

**Figure 2:**
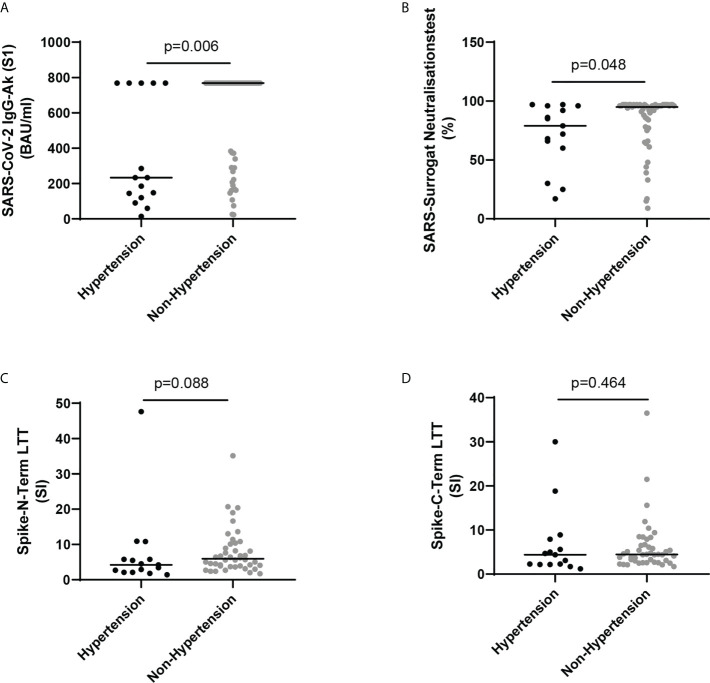
**(A)** SARS-CoV-2 IgG-Ab (S1) (BAU/ml) in vaccinated health care workers (HCWs) with and without hypertension. **(B)** SARS surrogate neutralization test (%) in vaccinated HCWs with and without hypertension. **(C)** Spike-N-Term LTT (SI) in vaccinated HCWs with and without hypertension. **(D)** Spike-C-Term LTT (SI) in vaccinated HCWs with and without hypertension. Lines show median. Comparison was made by Mann-Whitney U test.

Analyzing the humoral and cellular immune system in fully vaccinated HCWs - on the other side - showed elevated humoral response to SARS CoV-2, both SARS-CoV-2 IgG-Ab (S1) and SARS CoV-2 surrogate neutralization test, in the vaccinated non-hypertensive HCWs as compared to the vaccinated hypertensive HCWs, whereas the cellular response, both LTTs did not show differences between vaccinated hypertensive and not-hypertensive HCWs (**Figure 2** and **Table 2**).

### Multivariate regression analysis

Multivariate regression analysis revealed that hypertension was independently associated with humoral responses to SARS CoV-2 infection in the HCWs (**Table 5** and **Supplementary Tables 1A, B**) and showed a clear trend for hypertension being associated with the cellular response to SARS CoV-2 infection (**Table 5** and **Supplementary Tables 1C, D**). However, in vaccinated HCWs, hypertension was not significantly associated with any of the humoral and cellular response parameters in multivariate regression analysis (**Table 6** and **Supplementary Tables 2A–D**).

**Table 5:** Wild bootstrapping multivariate regression of hypertension with humoral and cellular response parameters as dependent variables in infected health care workers.

**Cellular and humoral response parameters**	**B**	**Bias**	**Std. Error**	**Sig.**	**BCa 95% Confidence Interval**	
					**Lower**	**Upper**
SARS-CoV-2 IgG-Ab (S1) (BAU/ml) ^a^	238.119	-5.462	80.72	0.013	91.835	376.501
SARS surrogate neutralization test (%) ^b^	16.544	-.891	6.747	0.020	4.892	26.704
Spike-N-Term LTT (SI) ^c^	6.599	0.062	3.153	0.078	0.325	12.943
Spike-C-Term LTT (SI) ^d^	4.792	0.058	2.456	0.066	0.045	9.805

The multiple regression models were performed with humoral and cellular response parameters as one dependent variable, respectively, i.e., SARS-CoV-2 IgG-Ab (S1) (BAU/ml) in model a, and R^2^ of the model is 0.255; SARS surrogate neutralization test (%) in model b, and R^2^ of the model is 0.194; Spike-N-Term LTT (SI) in model c, and R^2^ of the model is 0.304; Spike-C-Term LTT (SI) in model d, and R^2^ of the model is 0.260. We include confounding factors such as age (years), sex (M/F), BMI (body mass index, calculated as weight in kilograms divided by height in meters squared), diabetes (yes/no), hypertension (yes/no), chronic obstructive pulmonary disease (COPD) (yes/no), asthma (yes/no), smoking (yes/no), time from infection to blood collection (days) into the models. BCa, Bias-corrected and accelerated. Details of the individual models are shown in **Supplementary Table 1**.

**Table 6:** Wild bootstrapping multivariate regression of hypertension with humoral and cellular response parameters as dependent variables in vaccinated health care workers.

**Cellular and humoral response parameters**	**B**	**Bias**	**Std. Error**	**Sig.**	**BCa 95% Confidence Interval**	
					**Lower**	**Upper**
SARS-CoV-2 IgG-Ab (S1) (BAU/ml) ^a^	-55.970	1.539	123.554	0.703	-307.785	191.153
SARS surrogate neutralization test (%) ^b^	6.722	0.292	9.690	0.533	-11.061	25.611
Spike-N-Term LTT (SI) ^c^	1.109	0.142	3.742	0.838	-6.060	8.434
Spike-C-Term LTT (SI) ^d^	1.264	0.013	2.890	0.727	-4.129	6.811

The multiple regression models were performed with humoral and cellular response parameters as one dependent variable, respectively, i.e., SARS-CoV-2 IgG-Ab (S1) (BAU/ml) in model a, and R^2^ of the model is 0.285; SARS surrogate neutralization test (%) in model b, and R^2^ of the model is 0.227; Spike-N-Term LTT (SI) in model c, and R^2^ of the model is 0.114; Spike-C Term LTT (SI) in model d, and R^2^ of the model is 0.135. We include confounding factors such as age (years), sex (M/F), BMI (body mass index, calculated as weight in kilograms divided by height in meters squared), diabetes (yes/no), hypertension (yes/no), chronic obstructive pulmonary disease (COPD) (yes/no), asthma (yes/no), smoking (yes/no), time from vaccination to blood collection (days) and the type of vaccinations into the models. BCa, Bias-corrected and accelerated. Details of the individual models are shown in **Supplementary Table 2**.

## Discussion

The aim of the study was to characterize factors that determine the long-term humoral and cellular response of the immune system after SARS CoV-2 infection in a well-defined population of HCWs from a hospital with a SARS CoV-2 outbreak. Factors such as age, BMI, sex, diabetes, hypertension, smoking, COPD, asthma and time from infection/vaccination to blood collection were taken into account. The examination of the study participants at a median of 7 months after infection showed that humoral and probably cellular immunity after SARS Cov-2 infection were determined by pre-existing hypertension. In hypertensives, humoral and probably cellular immune responses were enhanced after infection. The hypertension effect was independent of factors such as age, BMI, sex, diabetes, smoking, COPD, asthma and time after infection.

In other words, our study clearly showed that the long-term response of the immune system to SARS CoV-2 infection is significantly influenced by the presence of hypertension. Interestingly, this association between hypertension and immune response was not found in vaccinated HCWs, and this is consistent with a previous study, which reported that BMI and hypertension are not associated with different immune responses in vaccinated HCWs (11). So far, only the opposite scientific question has been studied well i.e., the role of the immune system and its impact on blood pressure regulation. In the past years, the involvement of both the innate and adaptive immune system in the pathogenesis of hypertension has been established (12). The concept that the immune system has effects on blood pressure was established more than 50 years ago by Grollman et al. (13, 14) demonstrating that immunosuppression blunted hypertension in a model of renal infarction and that transfer of lymphocytes from rats with renal infarction induced hypertension in non-hypertensive animals. Later, it was shown that mice lacking adaptive immune cells, including recombinase-activating gene-deficient mice and rats, and mice with severe combined immunodeficiency have blunted blood pressure responses to classical stimuli causing hypertension such as ANG II, high salt, and norepinephrine (12, 15).

For SARS CoV-2 infections it was shown that hypertension is a key risk factor for poor outcome (16–19). A recent study by Trump et al. (2) using clinical data and single-cell RNA sequencing data of airway samples with in vitro experiments provided good evidence that high blood pressure delays viral clearance and exacerbates airway hyperinflammation in patients with SARS CoV-2 infection. The authors suggested that this might at least partially explain why hypertension is an independent risk factor for poor clinical outcome of COVID-19 patients what is in line with our findings that infected hypertensive HCWs were hospitalized likewise more frequently. The hypertension related delayed SARS CoV-2 clearance and exacerbated airway hyperinflammation may likewise explain our findings that hypertension is the only significant clinical risk factor associated with enhanced long-term immune response of the immune system to SARS CoV-2 infection. It is, however, important to note that the enhanced long-term stimulation of the immune response is specific to the SARS CoV-2 infection of the airways in hypertensive patients, because it was not observed after SARS CoV-2 vaccination (**Figures 1**, **2**; **Tables 5**, **6**), The hypertension specific alterations of the local immune system in the airways seems to be a prerequisite for the enhanced humoral and cellular immune response. If the contact of viral antigens (spike protein in case of vaccination) with the human body takes not place in the airway system, the answer of the immune system seems to be less pronounced. This hypothesis fits to the observations in the vaccinated HCWs (**Tables 2**, **6**, and **Figure 2**), After vaccination with a mRNA vaccine the immune system is stimulated by the expressed spike protein on the muscles where the vaccine was injected and the thereafter circulating spike protein in the blood.

The hypertension specific airway alteration in the response to SARS CoV-2 infection - delayed viral clearance and exacerbates airway hyperinflammation in hypertensive patients - that were recently described by Trump et al. (2) and Landmesser et al. (18) are obviously less important when the antigen (spike protein) circulates in the blood or is expressed on muscle cells where the vaccine was originally injected. Of course, a potential pathophysiological role of other viral components such as NC protein or M protein cannot be excluded.

It is furthermore of note that the long-term humoral and cellular response is independent of the symptoms after infection.

Since it has been suggested that an altered expression of genes in immune and epithelial cells typically seen in hypertensive patients is responsible for the augmented immune response in hypertensive SARS-CoV-2-positive COVID-19 patients (20), it thus would be of interest to see, whether treatment of hypertensive SARS-CoV-2 infected patients with dexamethasone, that will blunt the exacerbated airway hyperinflammation in patients with SARS Cov-2 infection and was clinically proven to improve outcome in complicated COVID-19 patients (21) will also result in a reduced long-term humoral and cellular response.

It is an obvious strength of our study that we were able to analyses a well-controlled study population getting twice weekly nasal/oral swabs for SARS-CoV-2 PCR testing and not being vaccinated. This ensures that we know exactly the time of infection (PCR positivity during systematic observation over 14 months). It is likewise a strength of the study that we applied wild bootstrapping multivariate regression analysis, which does not require homoskedasticity and considers potential bias. Given the nowadays good availability of vaccines such well controlled cohorts are very hard to establish. On the other hand, there are also limitations such as being a single center study with a middle-aged population. Since age is a key risk factor for poor COVID-19 outcome and immune response, long-term responses in elderly SARS CoV-2 infected hypertensive patients would be of interest. Moreover, the viral load at the infection points and some of treatment data concerning hypertension are missing in infected participants.

It is of note that hypertension exacerbates airway hyperinflammation in patients with COVID-19 and that treatment with ACE inhibitors might ameliorate airway hyperinflammation (2). Airway hyperinflammation was suggested to play a key pathophysiological role for COVID-19 disease severity and hence mortality. This might at least partially explain the clinical beneficial effects of ACEi/ARB treatment of hospitalized COVID-19 patients on mortality (16, 22–25).

In conclusion, our study demonstrated that SARS CoV-2 infection - even with mild or no symptoms - led to a long-lasting stimulation of the humoral and cellular immune system, probably because hypertension specifically delays viral clearance and exacerbates airway hyperinflammation.

## Data availability statement

The original contributions presented in the study are included in the article/**Supplementary Material**. Further inquiries can be directed to the corresponding authors.

## Ethics statement

The studies involving human participants were reviewed and approved by the ethical committee of the association of physicians in the province of Brandenburg, Germany. The patients/participants provided their written informed consent to participate in this study.

## Author contributions

BH and SE contributed to the conception and design of the study. AS, VvB and KK processed samples and performed laboratory measurements. CC and KK contributed to the data collection. CC and AS analyzed the results and composed all figures and tables. AS, CC, KK, and BH drafted the manuscript. BH, SE, and BKK contributed to the supervision of the analysis and revised the manuscript. All authors took part in the interpretation of the results and approved the final manuscript.

## Acknowledgment

China Scholarship Council supported CC.

## Conflict of interest

The authors declare that the research was conducted in the absence of any commercial or financial relationships that could be construed as a potential conflict of interest.

## Publisher’s note

All claims expressed in this article are solely those of the authors and do not necessarily represent those of their affiliated organizations, or those of the publisher, the editors and the reviewers. Any product that may be evaluated in this article, or claim that may be made by its manufacturer, is not guaranteed or endorsed by the publisher.
