# Supplementary material for: Impact of hypertension on long-term humoral and cellular response to SARS-CoV-2 infection
Source: Front Immunol. 2022 Sep 2;13:915001. doi: 10.3389/fimmu.2022.915001 (PMC9478933; doi:10.3389/fimmu.2022.915001)
Supplement: Supplementary file 1 [file Table_1.docx]

**Supplementary table 1(a).** Multivariate linear regression with wild bootstrapping for coefficients of SARS-CoV-2 IgG-Ab (S1) (BAU/ml) in infected health care workers.

| Model | B | Bias | Std. Error | Sig. | BCa 95% Confidence Interval | |
| --- | --- | --- | --- | --- | --- | --- |
|  |  |  |  |  | Lower | Upper |
| (Constant) | 183.964 | -12.698 | 192.365 | 0.389 | -183.878 | 525.364 |
| Age (years) | -0.554 | 0.112 | 2.206 | 0.824 | -4.647 | 3.87 |
| Sex (M/F) | -9.004 | 3.901 | 57.493 | 0.891 | -111.397 | 116.156 |
| BMI | -0.249 | 0.044 | 6.473 | 0.978 | -12.988 | 11.98 |
| Diabetes (yes/no) | -16.191 | 14.335 | 124.298 | 0.918 | -281.043 | 273.066 |
| Hypertension (yes/no) | 238.119 | -5.462 | 80.72 | 0.013 | 91.835 | 376.501 |
| COPD (yes/no) | 410.229 | -10.057 | 119.024 | 0.002 | 199.828 | 613.364 |
| Asthma (yes/no) | 25.899 | -2.387 | 77.853 | 0.774 | -119.094 | 171.874 |
| Smoking (yes/no) | -200.47 | -0.932 | 80.708 | 0.019 | -356.691 | -40.258 |
| Time from infection to blood collection (days) | 0.143 | 0.006 | 0.176 | 0.465 | -0.213 | 0.487 |

BCa, Bias-corrected and accelerated. Body mass index (BMI) was calculated as weight in kilograms divided by height in meters squared. COPD, chronic obstructive pulmonary disease. R^2^ of the model is 0.255.

**Supplementary table 1(b).** Multivariate linear regression with wild bootstrapping for coefficients of SARS surrogate neutralization test (%) in infected health care workers.

| Model | B | Bias | Std. Error | Sig. | BCa 95% Confidence Interval | |
| --- | --- | --- | --- | --- | --- | --- |
|  |  |  |  |  | Lower | Upper |
| (Constant) | 21.716 | -2.081 | 19.150 | 0.287 | -12.847 | 52.240 |
| Age (years) | 0.178 | 0.000 | 0.219 | 0.413 | -0.263 | 0.634 |
| Sex (M/F) | 5.779 | 0.434 | 5.723 | 0.345 | -5.679 | 17.940 |
| BMI | 0.536 | 0.035 | 0.577 | 0.395 | -0.643 | 1.750 |
| Diabetes (yes/no) | -16.314 | 3.271 | 12.541 | 0.272 | -43.002 | 15.678 |
| Hypertension (yes/no) | 16.544 | -.891 | 6.747 | 0.020 | 4.892 | 26.704 |
| COPD (yes/no) | 35.601 | -2.103 | 10.575 | 0.002 | 16.534 | 49.586 |
| Asthma (yes/no) | 6.515 | 0.542 | 6.840 | 0.373 | -7.852 | 21.918 |
| Smoking (yes/no) | -9.663 | -.929 | 6.566 | 0.167 | -21.531 | 0.524 |
| Time from infection to blood collection (days) | 0.023 | 0.002 | 0.018 | 0.237 | -0.015 | 0.062 |

BCa, Bias-corrected and accelerated. Body mass index (BMI) was calculated as weight in kilograms divided by height in meters squared. COPD, chronic obstructive pulmonary disease. R^2^ of the model is 0.194.

**Supplementary table 1(c).** Multivariate linear regression with wild bootstrapping for coefficients of T cell responses to SARS-CoV-2 Spike-N-Term (SI) in infected health care workers.

| Model | B | Bias | Std. Error | Sig. | BCa 95% Confidence Interval | |
| --- | --- | --- | --- | --- | --- | --- |
|  |  |  |  |  | Lower | Upper |
| (Constant) | 5.322 | 0.175 | 4.809 | 0.320 | -3.691 | 15.163 |
| Age (years) | 0.100 | 0.002 | 0.068 | 0.185 | -0.039 | 0.240 |
| Sex (M/F) | 0.297 | -0.038 | 1.623 | 0.892 | -2.652 | 3.485 |
| BMI | -0.104 | -0.009 | 0.155 | 0.551 | -0.397 | 0.179 |
| Diabetes (yes/no) | -3.330 | -0.106 | 3.172 | 0.390 | -9.253 | 2.280 |
| Hypertension (yes/no) | 6.599 | 0.062 | 3.153 | 0.078 | 0.325 | 12.943 |
| COPD (yes/no) | 1.515 | 0.145 | 4.842 | 0.773 | -7.166 | 11.924 |
| Asthma (yes/no) | 8.862 | 0.392 | 4.927 | 0.105 | -0.641 | 18.824 |
| Smoking (yes/no) | -1.325 | 0.107 | 1.237 | 0.347 | -3.889 | 1.408 |
| Time from infection to blood collection (days) | -0.007 | 0.000 | 0.010 | 0.619 | -0.024 | 0.010 |

BCa, Bias-corrected and accelerated. Body mass index (BMI) was calculated as weight in kilograms divided by height in meters squared. COPD, chronic obstructive pulmonary disease. R^2^ of the model is 0.304.

**Supplementary table 1(d).** Multivariate linear regression with wild bootstrapping for coefficients of T cell responses to SARS-CoV-2 Spike-C-Term (SI) in infected health care workers.

| Model | B | Bias | Std. Error | Sig. | BCa 95% Confidence Interval | |
| --- | --- | --- | --- | --- | --- | --- |
|  |  |  |  |  | Lower | Upper |
| (Constant) | 1.455 | 0.195 | 3.606 | 0.707 | -6.038 | 8.875 |
| Age (years) | 0.090 | 0.002 | 0.053 | 0.125 | -0.011 | 0.197 |
| Sex (M/F) | 0.902 | -0.045 | 1.241 | 0.522 | -1.306 | 3.149 |
| BMI | -0.075 | -0.001 | 0.099 | 0.485 | -0.274 | 0.110 |
| Diabetes (yes/no) | -4.093 | -0.458 | 1.820 | 0.062 | -6.945 | -2.073 |
| Hypertension (yes/no) | 4.792 | 0.058 | 2.456 | 0.066 | 0.045 | 9.805 |
| COPD (yes/no) | 0.298 | 0.175 | 1.643 | 0.878 | -3.127 | 4.092 |
| Asthma (yes/no) | 5.086 | -0.002 | 3.643 | 0.215 | -2.081 | 11.788 |
| Smoking (yes/no) | -0.494 | 0.104 | 0.752 | 0.543 | -2.092 | 1.343 |
| Time from infection to blood collection (days) | -0.001 | -0.001 | 0.006 | 0.832 | -0.013 | 0.009 |

BCa, Bias-corrected and accelerated. Body mass index (BMI) was calculated as weight in kilograms divided by height in meters squared. COPD, chronic obstructive pulmonary disease. R^2^ of the model is 0.260.

**Supplementary table 2(a).** Multivariate linear regression with wild bootstrapping for coefficients of SARS-CoV-2 IgG-Ab (S1) (BAU/ml) in vaccinated health care workers.

| Model | B | Bias | Std. Error | Sig. | BCa 95% Confidence Interval | |
| --- | --- | --- | --- | --- | --- | --- |
|  |  |  |  |  | Lower | Upper |
| (Constant) | 1269.508 | -3.500 | 211.974 | 0.001 | 872.337 | 1666.508 |
| Age (years) | -0.863 | 0.030 | 2.758 | 0.784 | -5.993 | 4.583 |
| Sex (M/F) | -35.166 | -1.040 | 61.657 | 0.624 | -156.243 | 84.988 |
| BMI | -7.547 | -0.039 | 7.167 | 0.334 | -23.182 | 7.106 |
| Diabetes (yes/no) | -110.890 | 4.455 | 172.245 | 0.599 | -445.352 | 225.141 |
| Hypertension (yes/no) | -55.970 | 1.539 | 123.554 | 0.703 | -307.785 | 191.153 |
| Asthma (yes/no) | -24.178 | -1.585 | 107.788 | 0.830 | -229.964 | 189.686 |
| Smoking (yes/no) | -74.366 | 5.555 | 72.917 | 0.346 | -232.489 | 95.126 |
| Time from vaccination to blood collection (days) | -2.489 | 0.028 | 0.916 | 0.017 | -4.303 | -0.582 |
| Type of vaccinations | -109.009 | -0.186 | 47.389 | 0.040 | -201.418 | -20.687 |

BCa, Bias-corrected and accelerated. Body mass index (BMI) was calculated as weight in kilograms divided by height in meters squared. Chronic obstructive pulmonary disease (n=0). R^2^ of the model is 0.285.

**Supplementary table 2(b).** Multivariate linear regression with wild bootstrapping for coefficients of SARS surrogate neutralization test (%) in vaccinated health care workers.

| Model | B | Bias | Std. Error | Sig. | BCa 95% Confidence Interval | |
| --- | --- | --- | --- | --- | --- | --- |
|  |  |  |  |  | Lower | Upper |
| (Constant) | 136.993 | 0.901 | 18.405 | 0.001 | 100.214 | 176.485 |
| Age (years) | -0.279 | -0.005 | 0.219 | 0.248 | -0.720 | 0.123 |
| Sex (M/F) | -8.143 | -0.033 | 5.003 | 0.145 | -18.327 | 1.222 |
| BMI | -0.332 | -0.012 | 0.583 | 0.610 | -1.421 | 0.804 |
| Diabetes (yes/no) | -22.632 | -0.561 | 15.911 | 0.198 | -53.785 | 8.430 |
| Hypertension (yes/no) | 6.722 | 0.292 | 9.690 | 0.533 | -11.061 | 25.611 |
| Asthma (yes/no) | 0.913 | -0.032 | 8.736 | 0.937 | -16.280 | 18.484 |
| Smoking (yes/no) | -11.285 | 0.137 | 6.915 | 0.139 | -24.632 | 2.540 |
| Time from vaccination to blood collection (days) | -0.123 | -0.001 | 0.064 | 0.094 | -0.258 | 0.009 |
| Type of vaccinations | -5.135 | -0.265 | 3.406 | 0.188 | -11.496 | 0.928 |

BCa, Bias-corrected and accelerated. Body mass index (BMI) was calculated as weight in kilograms divided by height in meters squared. Chronic obstructive pulmonary disease (n=0). R^2^ of the model is 0.227.

**Supplementary table 2(c).** Multivariate linear regression with wild bootstrapping for coefficients of SARS-CoV-2 Spike-N-Term (SI) in vaccinated health care workers.

| Model | B | Bias | Std. Error | Sig. | BCa 95% Confidence Interval | |
| --- | --- | --- | --- | --- | --- | --- |
|  |  |  |  |  | Lower | Upper |
| (Constant) | 10.289 | 0.369 | 5.549 | 0.109 | -1.285 | 22.530 |
| Age (years) | 0.016 | 0.000 | 0.071 | 0.863 | -0.110 | 0.156 |
| Sex (M/F) | -1.968 | -0.068 | 2.428 | 0.512 | -6.483 | 2.396 |
| BMI | -0.287 | -0.013 | 0.209 | 0.243 | -0.661 | 0.068 |
| Diabetes (yes/no) | -3.939 | 0.054 | 4.809 | 0.497 | -12.765 | 5.192 |
| Hypertension (yes/no) | 1.109 | 0.142 | 3.742 | 0.838 | -6.060 | 8.434 |
| Asthma (yes/no) | -0.573 | -0.068 | 3.325 | 0.903 | -7.223 | 5.438 |
| Smoking (yes/no) | 0.453 | -0.194 | 3.216 | 0.921 | -5.329 | 5.866 |
| Time from vaccination to blood collection (days) | 0.037 | 0.002 | 0.028 | 0.278 | -0.014 | 0.092 |
| Type of vaccinations | 2.213 | 0.004 | 1.628 | 0.280 | -0.644 | 4.919 |

BCa, Bias-corrected and accelerated. Body mass index (BMI) was calculated as weight in kilograms divided by height in meters squared. Chronic obstructive pulmonary disease (n=0). R^2^ of the model is 0.114.

**Supplementary table 2(d).** Multivariate linear regression with wild bootstrapping for coefficients of SARS-CoV-2 Spike-C-Term (SI) in vaccinated health care workers.

| Model | B | Bias | Std. Error | Sig. | BCa 95% Confidence Interval | |
| --- | --- | --- | --- | --- | --- | --- |
|  |  |  |  |  | Lower | Upper |
| (Constant) | 6.468 | 0.836 | 5.516 | 0.304 | -5.175 | 19.710 |
| Age (years) | 0.070 | 0.002 | 0.067 | 0.408 | -0.047 | 0.196 |
| Sex (M/F) | -2.228 | -0.056 | 2.318 | 0.453 | -6.703 | 1.760 |
| BMI | -0.124 | -0.014 | 0.176 | 0.526 | -0.446 | 0.172 |
| Diabetes (yes/no) | -5.782 | 0.170 | 3.576 | 0.163 | -13.033 | 2.012 |
| Hypertension (yes/no) | 1.264 | 0.013 | 2.890 | 0.727 | -4.129 | 6.811 |
| Asthma (yes/no) | -1.764 | 0.108 | 2.692 | 0.562 | -7.487 | 3.570 |
| Smoking (yes/no) | 3.252 | -0.032 | 3.191 | 0.441 | -2.199 | 8.644 |
| Time from vaccination to blood collection (days) | 0.025 | -0.002 | 0.019 | 0.257 | -0.012 | 0.059 |
| Type of vaccinations | 0.639 | -0.145 | 1.187 | 0.652 | -1.539 | 2.500 |

BCa, Bias-corrected and accelerated. Body mass index (BMI) was calculated as weight in kilograms divided by height in meters squared. Chronic obstructive pulmonary disease (n=0). R^2^ of the model is 0.135.
